# Distinct mutational pattern of T-cell large granular lymphocyte leukemia combined with pure red cell aplasia: low mutational burden of *STAT3*

**DOI:** 10.1038/s41598-023-33928-z

**Published:** 2023-05-04

**Authors:** Sooyong Park, Jiwon Yun, Sung Yoon Choi, Dajeong Jeong, Ja-Yoon Gu, Jee-Soo Lee, Moon-Woo Seong, Yoon Hwan Chang, Hongseok Yun, Hyun Kyung Kim

**Affiliations:** 1 Department of Laboratory Medicine, Seoul National University College of Medicine, Seoul, Republic of Korea; 2 Department of Laboratory Medicine, Chung-Ang University College of Medicine, Seoul, Republic of Korea; 3 Cancer Research Institute, Seoul National University College of Medicine, Seoul, Republic of Korea; 4 Department of Genomic Medicine, Seoul National University Hospital, Seoul, Korea

**Keywords:** Haematological cancer, Cancer genomics

## Abstract

T-cell large granular lymphocyte leukemia (T-LGL) is often accompanied by pure red cell aplasia (PRCA). A high depth of next generation sequencing (NGS) was used for detection of the mutational profiles in T-LGL alone (n = 25) and T-LGL combined with PRCA (n = 16). Beside *STAT3* mutation (41.5%), the frequently mutated genes included *KMT2D* (17.1%), *TERT* (12.2%), *SUZ12* (9.8%), *BCOR* (7.3%), *DNMT3A* (7.3%), and *RUNX1* (7.3%). Mutations of the *TERT* promoter showed a good response to treatment. 3 of 41 (7.3%) T-LGL patients with diverse gene mutations were revealed as T-LGL combined with myelodysplastic syndrome (MDS) after review of bone marrow slide. T-LGL combined with PRCA showed unique features (low VAF level of *STAT3* mutation, low lymphocyte count, old age). Low ANC was detected in a *STAT3* mutant with a low level of VAF, suggesting that even the low mutational burden of *STAT3* is sufficient for reduction of ANC. In retrospective analysis of 591 patients without T-LGL, one MDS patient with *STAT3* mutation was revealed to have subclinical T-LGL. T-LGL combined with PRCA may be classified as unique subtype of T-LGL. High depth NGS can enable sensitive detection of concomitant MDS in T-LGL. Mutation of the *TERT* promoter may indicate good response to treatment of T-LGL, thus, its addition to an NGS panel may be recommended.

## Introduction

T-cell large granular lymphocyte leukemia (T-LGL) is a rare mature neoplasm of T cells induced by clonal expansion of large granular T lymphocytes, usually presenting with neutropenia^1^. Autoimmune diseases are often associated with T-LGL in Western countries, while pure red cell aplasia (PRCA) is more often reported in Asian countries^2,3^.

PRCA is characterized by anemia and severe erythroid hypoplasia in bone marrow (BM)^4^. Development of PRCA occurs along with various underlying diseases such as thymoma, T-LGL, and autoimmune diseases. It is also a bone marrow failure syndrome (BMFS), defined by failure to produce adequate hematopoietic cells in BM, along with aplastic anemia and myelodysplastic syndrome (MDS). BMFS is characterized by immune attack on stem cells via a T-cell mediated immune mechanism and expansion of T cells in BM has been detected^5,6^. It has not yet been determined whether expansion of T cells is simply a response to immune attack or clonal selection^6^.

The discovery of somatic mutations of *STAT3* has led to a more optimistic outlook regarding the genetic basis of T-LGL pathogenesis^7^. Activation of *STAT3* induced by somatic mutation is thought to facilitate proliferation of T cells through enhanced transcription of anti-apoptotic proteins and downregulation of TP53^8,9^. Although specificity of *STAT3* mutations for T-LGL has been reported^10^, it has also been reported in other conditions showing T cell expansion including PRCA, aplastic anemia, MDS, and other autoimmune disorders^11,12^. Detection of *STAT5B* mutations has been reported in 2% of cases of T-LGL, particularly in association with CD4 + T-LGL^13,14^. Despite frequent detection of *STAT3* and *STAT5B* mutations in T-LGL, their clinical specificity for diagnosis of T-LGL has not been elucidated.

Although two whole-exome sequencing studies have reported on somatic mutation of genes other than *STAT3* in T-LGL^15,16^, the two studies reported significantly different mutational spectrums. Because mutation of clonal hematopoiesis-related genes including *TET2, DNMT3A*, and *BCOR* often occurs in BMFS such as PRCA and aplastic anemia^17,18^ and a subset of T-LGL occurs concomitantly with BMFS^6,12,19,20^, the mutational patterns of T-LGL may differ according to the concomitant disorders. The significant variation in frequency of *STAT3* mutation in PRCA reported in the literature, ranging from 0 to 63%, appears to depend on concomitant T-LGL^21,22^.

In this study, high depth of next generation sequencing (NGS) of 84 candidate genes including all genes detected in PRCA or aplastic anemia^7,17,18,23–25^ was performed in order to examine the mutational patterns in T-LGL with or without PRCA. Conduct of the study demonstrated the difference in mutational patterns according to the concomitant PRCA, *STAT3* mutation, or treatment response and the association of mutational extent of *STAT3* with clinical features. We also examined the frequency of *STAT3* and *STAT5B* mutation in hematologic diseases without T-LGL.

## Results

### Patient characteristics

The median age was higher for patients with T-LGL combined with PRCA (T-LGL + PRCA) compared to those with T-LGL alone (Table 1). Significantly lower levels of hemoglobin and absolute reticulocyte count were detected in patients with T-LGL + PRCA compared to those with T-LGL. In addition, a higher absolute lymphocyte count (ALC) was detected in patients with T-LGL compared to those with T-LGL + PRCA, while a lower absolute neutrophil count (ANC) was detected in patients with T-LGL. There was no difference in platelet count. T-LGL + PRCA showed significantly higher RBC transfusion dependency compared to T-LGL. A higher percentage of patients with T-LGL had a history of infection compared to those with T-LGL + PRCA, but without statistical significance.

**Table 1:** Characteristics of the study population.

	PRCA (n = 3)	T-LGL (n = 25)	T-LGL + PRCA (n = 16)	*P*-value^↑^
Age (years)	53 (50.5–64.5)	55 (45–70)	71 (56–77)	0.048
Male/Female (number)	1/2	11/14	6/10	0.680
Autoimmune diseases* (number)	0	2 (8.0%)	1 (6.3%)	1.000
Hemoglobin (g/dL)	7.2 (6.2–7.4)	12.8 (9.2–14.0)	6.7 (5.7–7.6)	< 0.001
Absolute reticulocyte count (× 10^9^/L)	7.2	51.1 (42.9–71.6)	16.9 (5.8–23.5)	< 0.001
Absolute lymphocyte count (× 10^9^/L)	0.8 (0.7–1.3)	3.9 (2.3–6.2)	1.0 (0.7–2.1)	< 0.001
Absolute neutrophil count (× 10^9^/L)	4.2 (3.5–5.2)	1.1 (0.5–1.3)	1.3 (1.0–2.0)	0.040
Platelet count (× 10^9^/L)	203 (192–252)	213 (149–272)	217 (170–360)	0.521
RBC transfusion dependency (number)	3 (100%)	2 (8.0%)	9 (56.3%)	0.012
Infection history (number)	1 (33.3%)	10 (40.0%)	3 (18.8%)	0.187
Treatment received (number)	2 (66.6%)	18 (72.0%)	13 (81.3%)	0.713
Bone marrow cellularity (%)	25.0 (25.0–35.0)	45.0 (31.3–65.0)	40.0 (25.0–55.0)	0.405
*STAT3* mutation (number)	0	9 (36.0%)	8 (50.0%)	0.280
*STAT3* VAF (%)	–	24.8 (22.1–29.4)	4.5 (3.5–6.6)	< 0.001

The values are presented as the median value or number and interquartile range or percentage in parentheses.

*Comorbid autoimmune diseases included Behcet’s disease and systemic lupus erythematosus in T-LGL and rheumatic arthritis in T-LGL + PRCA.

^↑^*P*-values compare T-LGL with T-LGL + PRCA.

*PRCA*, pure red cell aplasia; *T-LGL*, T-cell large granular leukemia; *T-LGL + PRCA*, T-LGL combined with PRCA; *STAT3*, signal transducer and activator of transcription 3; *VAF*, variant allele frequency.

### Mutational landscape in PRCA, T-LGL and T-LGL + PRCA

**Figure 1:**
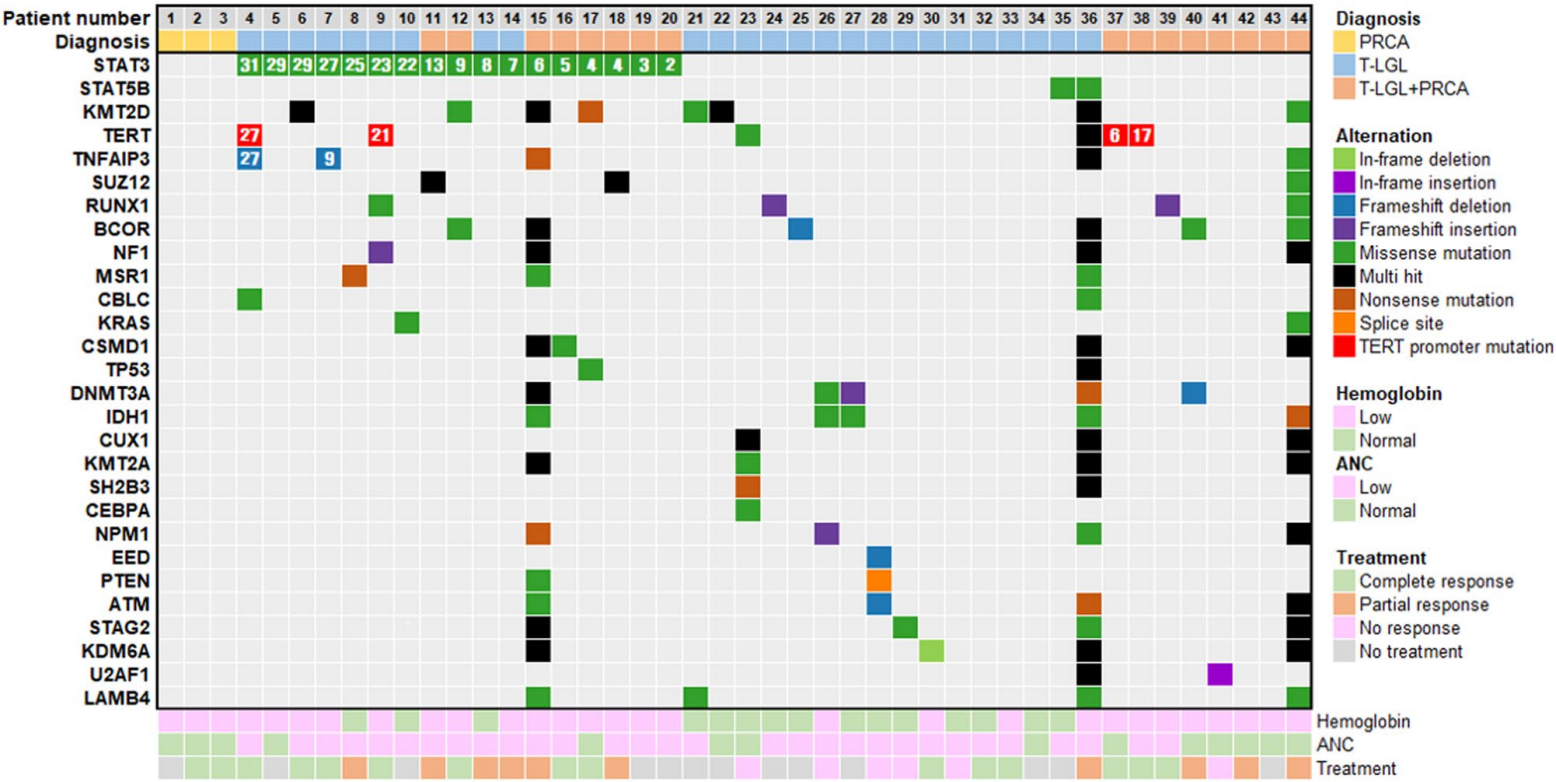
Oncoplot of targeted gene mutations in pure red cell aplasia (PRCA, n = 3), T-cell large granular lymphocyte leukemia (T-LGL, n = 25) and T-LGL combined with PRCA (T-LGL + PRCA, n = 16). Among 84 genes tested, 28 genes in which one or more potential somatic mutations were detected were shown. The genes that were not detected any mutation were removed and are shown in Supplementary Table 5. In *STAT3* mutation, variant allele fraction (VAF) percentage was shown in numbers and arranged in order of VAF (%). In some other mutations, the VAF was shown in numbers. Bottom annotations represent clinical parameters at diagnosis and treatment responses. Lower than 10 g/dL of hemoglobin was marked as “Low” and if it was higher than 10 g/dL, marked as “Normal”. Absolute neutrophil count (ANC) cut-off value was 1.8 × 10^9^/L. Conventional cytogenetic results showed a normal karyotype in 42 patients, except for two patients (Y chromosome loss, 26.1% in patient number 9 and 20.0% in patient number 17). Treatment response definitions are described in the “Methods” section.

No mutation was detected in patients with PRCA (n = 3) (Fig. 1). Of 41 patients with T-LGL or T-LGL + PRCA, *STAT3* mutation was detected in 17 patients (41.5%), nine (36.0%) of 25 patients with T-LGL, and eight (50.0%) of 16 patients with T-LGL + PRCA (Table 1 and Fig. 1). When aligning with the level of *STAT3* VAF, a low level of VAF (< 10.0%) was detected in only two patients with T-LGL (22.2%, 2/9) compared to those with T-LGL + PRCA (87.5%, 7/8, *P* = 0.015, Fig. 1). *STAT5B* mutations were detected in two patients with *STAT3* wild type.

Mutations of diverse genes were detected in three patients (patient numbers 15, 36, 44) with VAF levels of 2~3% (Supplementary Table 2). Following review of BM slides, the three patients were additionally diagnosed with MDS combined with T-LGL (Supplementary Table 3). The three patients showed no abnormal cytogenetic results.

Excluding the three patients described above with a concomitant MDS clone, 65 mutations were detected in 28 genes (33.3%) of all 84 targeted genes in 32 patients with T-LGL or T-LGL + PRCA (Fig. 1 and Supplementary Table 4) and no mutation was detected in 56 genes (Supplementary Table 5). After excluding *STAT3* mutations, the genes showing frequent mutation included *KMT2D* (17.1%), *TERT* (12.2%), *SUZ12* (9.8%), *BCOR* (7.3%), *DNMT3A* (7.3%), and *RUNX1* (7.3%). *TERT* mutations were detected in two *STAT3* mutant patients (c.-124C > T in 2) and in three *STAT3* wild type patients (c.-124C > T, c.-146C > T and c.81C > A). Among *STAT3* mutant patients, the levels of VAF detected in *TERT* were similar to those detected in *STAT3* in two patients with *TERT* promoter (patient numbers 4, 9).

Different mutational patterns were observed between *STAT3* mutant and wild type. Mutations of *SUZ12* (9.8%) and *TNFAIP3* (4.9%) were only detected in *STAT3* mutant, while mutations of *DNMT3A* (7.3%) and *IDH1* (4.9%) were only detected in *STAT3* wild type after excluding three patients with a concomitant MDS clone. *TNFAIP3* mutations (nonsense mutation L303* in patient number 4, frameshift R141A*75 in patient number 7) showed comparable or a lower level of VAF than *STAT3*. *NF1* and *KRAS* mutations were detected in both *STAT3* mutant with the same frequencies (2.4%). *TP53* mutation (patient number 17) showed comparable level of VAF to *STAT3*.

### Changes of CBC parameters in terms of *STAT3* mutations

**Figure 2:**
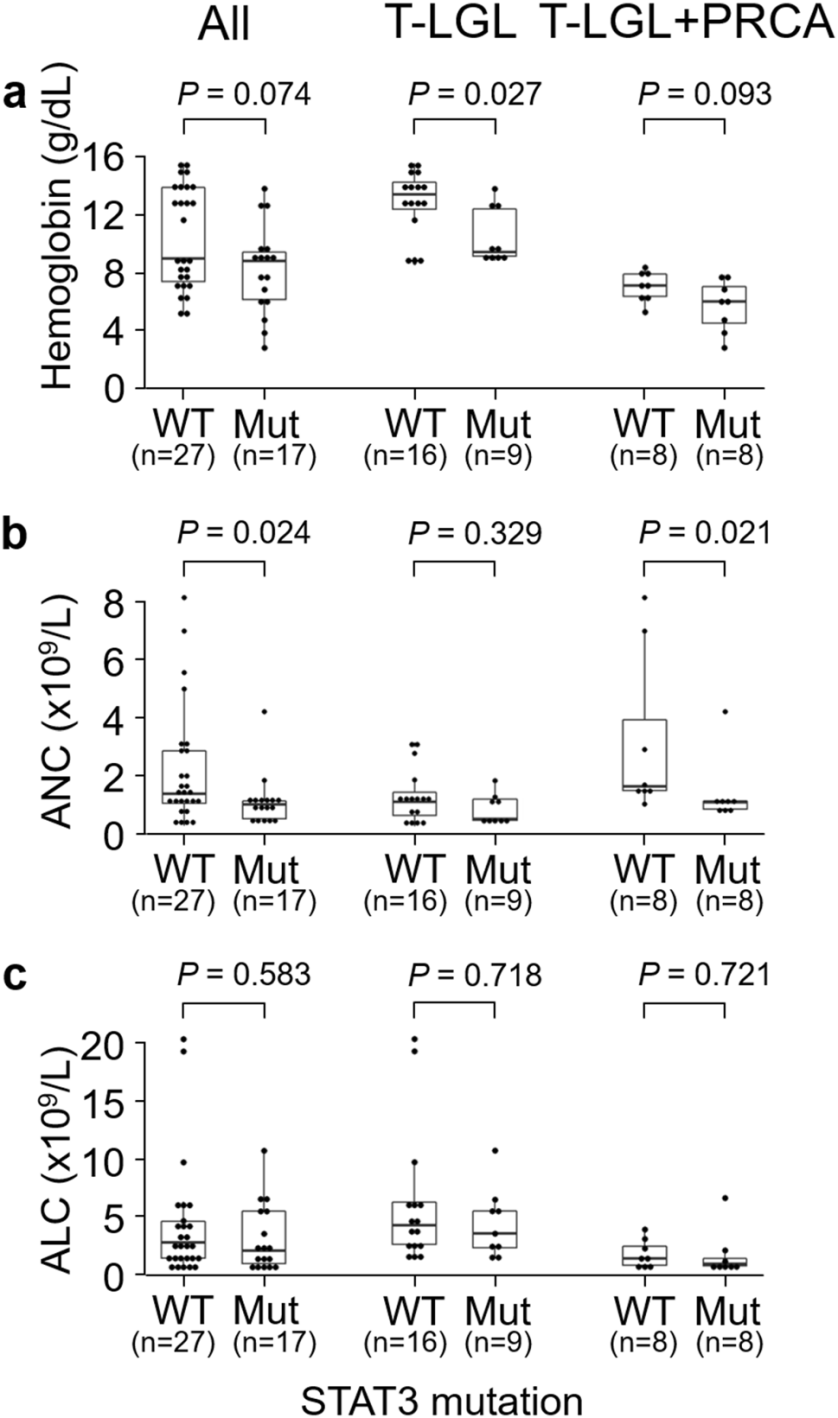
Changes of CBC parameters in terms of *STAT3* mutation. (**a**) The hemoglobin level, (**b**) absolute neutrophil count (ANC) and (**c**) absolute lymphocyte count (ALC) were compared between *STAT3* wild type (WT, n = 27) and *STAT3* mutant (Mut, n = 17) in all patients (n = 44). After they were divided into two subgroups, comparisons were performed between *STAT3* WT (n = 16) and *STAT3* Mut (n = 9) in T-LGL (n = 25) and between *STAT3* WT (n = 8) and *STAT3* Mut (n = 8) in T-LGL + PRCA (n = 16). Boxplots show the median values and interquartile ranges of CBC parameters and Wilcox rank sum tests were used for statistics. Abbreviations: PRCA, pure red cell aplasia; T-LGL, T-cell large granular lymphocyte leukemia; T-LGL + PRCA, T-LGL combined with PRCA; *STAT3*, signal transducer and activator of transcription 3.

A low level of hemoglobin was detected in *STAT3* mutant patients (n = 17) compared with *STAT3* wild type (n = 27) in all patients (n = 44), however statistical significance was not reached (Fig. 2a). When divided into subgroups, in T-LGL alone (n = 25), significantly lower levels of hemoglobin were detected for *STAT3* mutant compared with those of *STAT3* wild type. Significantly lower levels of ANC were detected in *STAT3* mutant compared with *STAT3* wild type in all patients (Fig. 2b). In the T-LGL + PRCA (n = 16), significantly low levels of ANC were detected in *STAT3* mutant patients. No statistical difference in ALC was observed between *STAT3* mutant patients and wild type patients across all three groups (Fig. 2c).

### Association of *STAT3* mutational burden with CBC parameters

**Figure 3:**
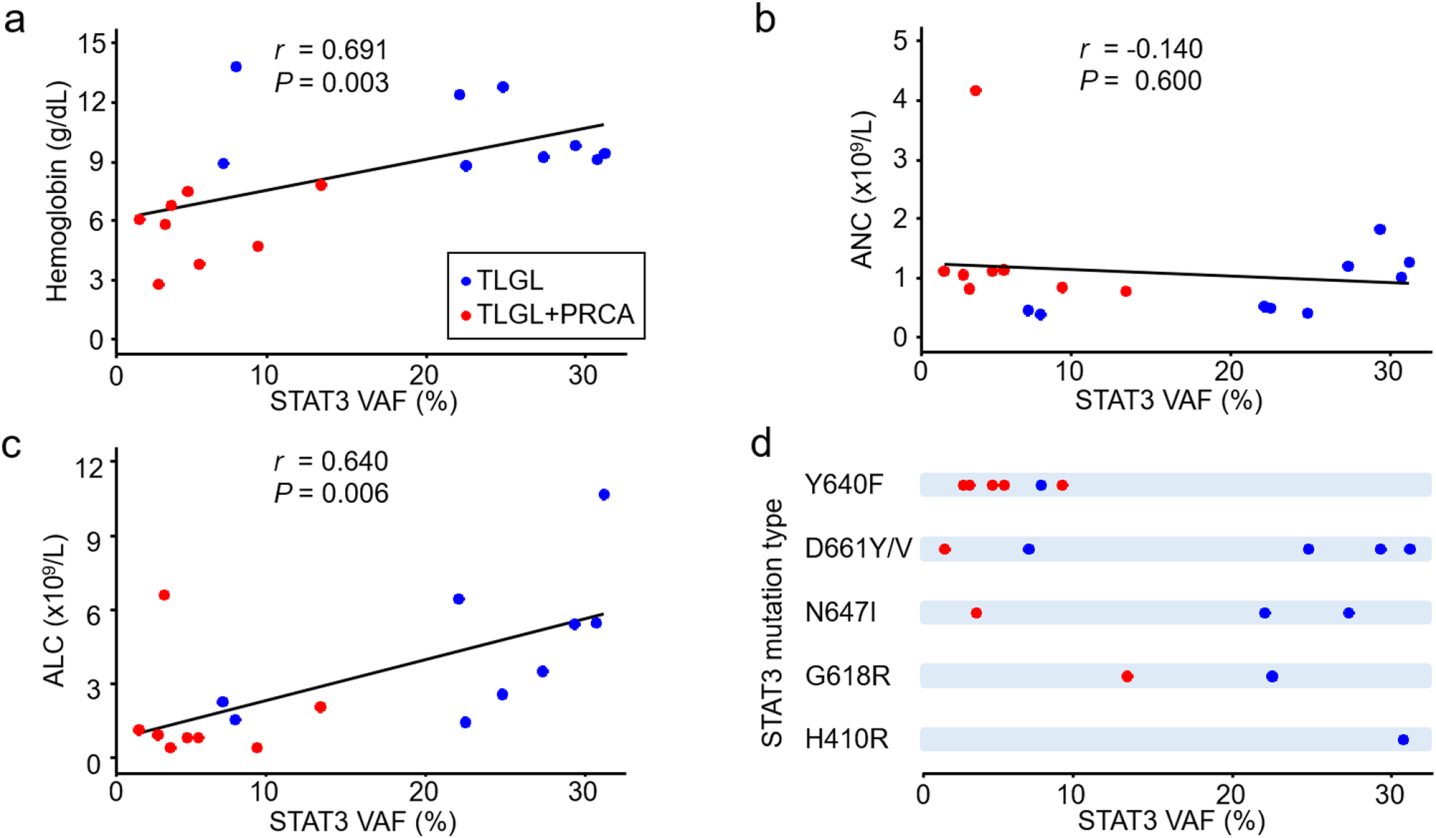
Association of *STAT3* mutational extent with CBC parameters. (**a**) The hemoglobin level, (**b**) absolute neutrophil count (ANC) and (**c**) absolute lymphocyte count (ALC) were presented with variant allele frequency (VAF) levels of *STAT3* mutation in *STAT3* mutant (n = 17). The red dots represent patients with T-LGL (n = 9) and blue dots represents patients with T-LGL + PRCA (n = 8). Linear lines are linear regression fits. (**d**) Mutation types of the *STAT3* gene were presented with *STAT3* VAF in order of detected frequency. Abbreviations: the same as shown in Fig. 2.

The levels of VAF detected in the *STAT3* mutation showed significant correlation with hemoglobin levels (Fig. 3a). Of note, seven patients (87.5%, 7/8) in the T-LGL + PRCA, marked by red dots, showed low (< 10%) levels of VAF, while only two patients (22.2%, 2/9) with T-LGL, marked by blue dots, showed low levels of VAF. A significantly lower median level of VAF was detected in *STAT3* in T-LGL + PRCA (4.5%, IQR 3.5–6.6%) compared with T-LGL (24.8%, 22.1–29.4%) (Table 1).

Among 17 *STAT3* mutations, 16 mutations were localized in the src homology 2 domain where Y640F (n = 6), D661Y/V (n = 5), N647I (n = 3), and G618R (n = 2) were detected (Fig. 3d, Supplementary Fig. 1). In the DNA-binding domain, one H410R mutation was detected in T-LGL. The most frequent mutation, Y640F, was mainly observed in low level VAF (< 10%) and prevalent in T-LGL + PRCA (62.5%, 5/8), which differed significantly from the prevalence in T-LGL (11.1%, 1/9, *P* = 0.043, Fig. 3d). Of particular interest, Y640F was the most commonly detected mutation among patients with < 10% VAF of *STAT3* mutation in our population (66.7%, 6/9) and in other reports (59.6%, 28/47)^26^ (Supplementary Fig. 2). ANC showed no correlation with the level of *STAT3* VAF (Fig. 3b), however ALC showed significant correlation with the level of *STAT3* VAF (Fig. 3c). Platelet count showed no correlation with *STAT3* VAF.

### Therapeutic effect according to mutations

CR or PR was achieved in 25 (80.6%) of 31 patients (Fig. 1). All *STAT3* mutant patients showed CR or PR to treatments (100%, 13/13), which was higher compared with *STAT3* wild type (66.6%, 12/18, *P* = 0.028). Of particular interest, patients with mutation of the *TERT* promoter (n = 4, marked in red in Fig. 1) showed CR (100%, 4/4), while 40.7% of patients without mutation of the *TERT* promoter showed CR (11/27, *P* = 0.043).

### Specificity of *STAT3* and *STAT5B* mutations in hematologic diseases without T-LGL

Conduct of a retrospective analysis of 591 hematologic patients without T-LGL resulted in identification of three patients with *STAT3* (n = 1, 0.17%) or *STAT5B* (n = 2, 0.34%) mutations. One patient with *STAT3* mutation (G618R, VAF 7.6%) had already been diagnosed with therapy-related MDS. Following performance of additional CD3, CD4, CD8, and granzyme B staining for reexamination of BM slides, increased interstitial infiltration of CD3, CD8 and granzyme B positive T cells was observed, demonstrating co-existence of T-LGL (ALC, 2.3 × 10^9^/L; Large granular lymphocyte, 1.4 × 10^9^/L). After all, because no *STAT3* mutation was detected in 590 patients who did not have T-LGL, *STAT3* mutation showed 100% specificity for diagnosis of T-LGL. Two patients with mutation of *STAT5B* were MDS-excess blasts-1 (N642H, VAF 4.8%) and idiopathic hypereosinophilia (V712E, VAF 7.9%) with no evidence of T cell infiltration.

## Discussion

**Figure 4:**
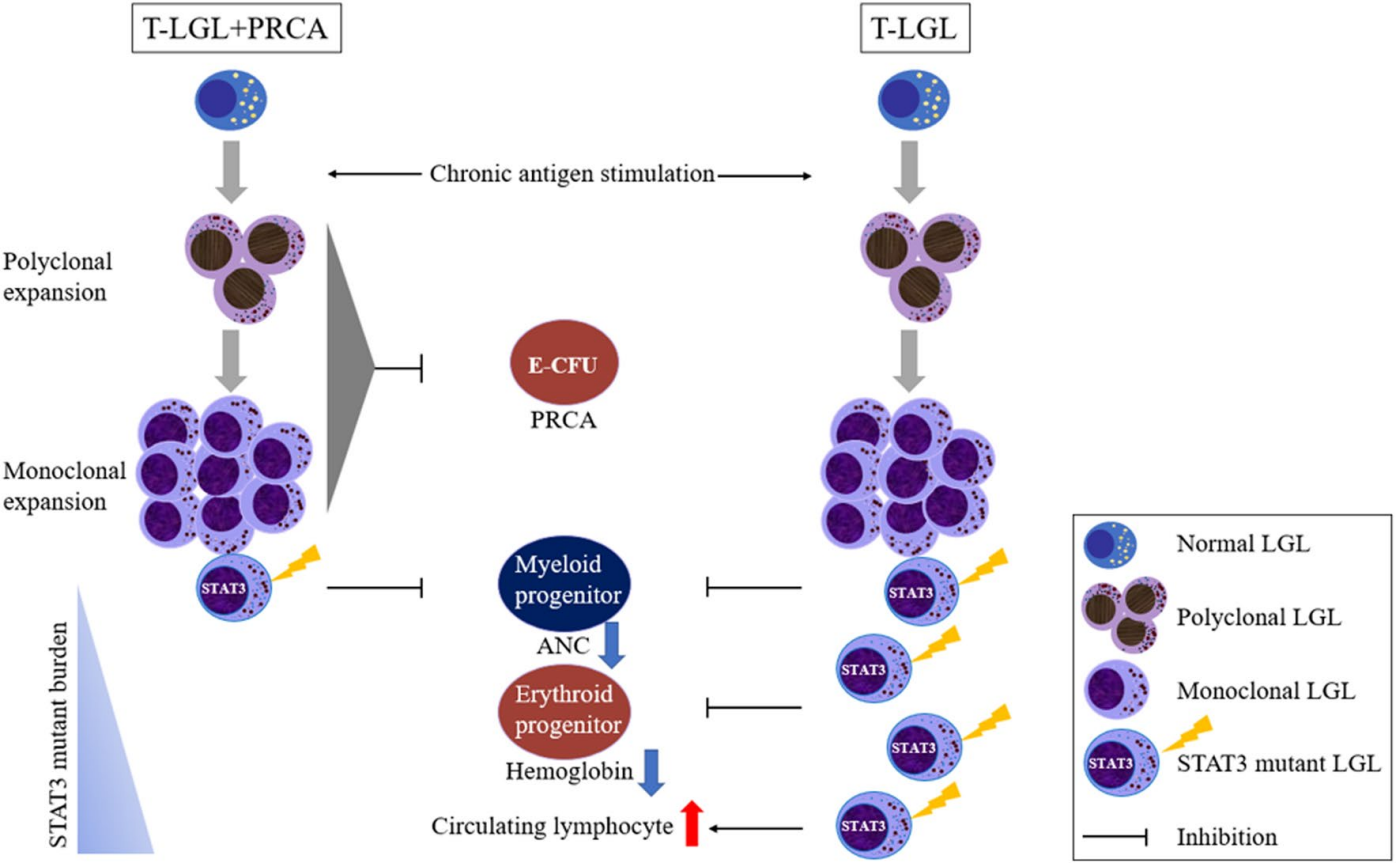
Proposed pathogenesis of T-cell large granular lymphocyte leukemia (T-LGL) combined with pure red cell aplasia (PRCA) (T-LGL + PRCA). Various stimuli including infection, cytokines, autoimmune, or unknown antigens cause polyclonal expansion of T large granular lymphocytes (LGL). The ongoing antigen stimulation can induce monoclonal expansion of LGL. The expanded polyclonal or monoclonal LGL is capable of inhibiting erythroid colony-forming units (E-CFU) and inducing PRCA. During monoclonal expansion, the *STAT3* mutations can occur or activation of *STAT3* occur through another non-mutational mechanism. Although there was no report proving a direct inhibitory effect of *STAT3* mutated T cells on myelopoiesis, our data suggest that low mutational burden of *STAT3* in T-LGL + PRCA may induce a greater reduction of absolute neutrophil count (ANC) than wild type. Of note, high mutational burden of *STAT3* observed in T-LGL alone may not only reduce ANC and hemoglobin, but also increase the circulating lymphocyte count. Conduct of further study of the mutational process of the *STAT3* gene and the functional role of *STAT3*-mutated T cells will be required in the future.

Mediation of idiopathic PRCA was originally considered to occur by way of autoreactive T cells capable of destroying erythroid colony-forming units by inhibition of erythropoiesis^27^. Expansion of T cells can be polyclonal or monoclonal with positive T cell receptor (TCR) rearrangement^28^. Activation of the *STAT3* pathway occurs during development of T cells for undertaken mutagenesis^29^. Mutation of *STAT3* may occur during the monoclonal expansion of T cells (Fig. 4). The occurrence of *STAT3* mutation can proffer a survival benefit through the gain-of-function mutation, inducing malignant transformation and subsequent development of T-LGL.

In the T-LGL + PRCA, the low levels of hemoglobin detected in the *STAT3* wild type were similar to those detected in the *STAT3* mutant (Fig. 2a), suggesting the existence of a severe anemic state regardless of emergence of *STAT3* mutation. In addition, the low level of ALC detected in patients with T-LGL + PRCA (Table 1) compared to those with T-LGL alone also suggests newly developing T-LGL in already existing PRCA. Beside the low levels of ALC, patients with T-LGL + PRCA were older in age, compared to those with T-LGL alone, implying that T-LGL + PRCA is a unique disease entity separate from T-LGL alone, which should be classified as a subtype of T-LGL.

Mutation of *STAT3* has been reported to occur in 30~75% of patients with T-LGL^6,15,16,30^, similar to our result of 41.5% in patients with T-LGL. Shi et al. reported that mutation of *STAT3* was not detected using Sanger sequencing in patients with T-LGL + PRCA^21^, while in our study, mutation of *STAT3* was detected using a high depth of sequencing in 50% of patients with T-LGL + PRCA, particularly with mainly low levels of VAF. This may be attributable to differences in the technical sensitivity of the type of sequencing used. We were able to determine the characteristics of T-LGL + PRCA, which usually showed a low level of VAF in the *STAT3* mutation resulting from use of high depth of sequencing. Use of a high depth of sequencing is important for detection of low mutant clones.

Since the lower limit of detection for Sanger sequencing is usually around 10%^26^, we arbitrarily defined the cut-off of *STAT3* mutation as 10%. Interestingly, the low level of VAF (< 10.0%) was mostly detected in T-LGL + PRCA. Moreover, *STAT3* Y640F mutation was the most common mutations in T-LGL + PRCA. These findings suggest T-LGL + PRCA is a unique disease entity that can be classified as a subtype of T-LGL. Jerez et al. suggested that *STAT3* mutant immune attack itself can cause primary induction of BMFS, which may mean even a low mutational burden of *STAT3* can influence disease progression^12^.

The VAF levels of mutated genes partly depend on the proportion of large granular lymphocytes in the analyzed samples. Therefore, the cell sorting has been tried to overcome the variation^7,31^. Using CD8 + T cell sorting technique, *STAT3* mutations were exclusively detected in certain TCR Vβ population of CD8 + T cells^31^. Since our study did not use the cell sorting technique, we could not adjust *STAT3* VAF levels according to the large granular lymphocyte counts. Recently, there has been a standardization approach in NGS that improve tumor purity metrics by using new computational algorithms for neoplastic cellularity^32^. It is worth applying the standardized approach to compare VAF levels in future study.

An association of *STAT3* mutation with a low level of ANC has been reported^16,33^. Similarly, our results for all cases of T-LGL showed that the level of ANC was lower in the *STAT3* mutant than in wild type. However, when divided into subgroups, a low level of ANC was detected in patients with T-LGL regardless of *STAT3* mutation (Fig. 2b), indicating that the low level of ANC in T-LGL was independent of *STAT3* mutation. Within patients with T-LGL + PRCA, *STAT3* mutant patients with usually low levels of VAF showed lower levels of ANC compared with *STAT3* wild type (Fig. 2b) and even patients with a high level of VAF in the *STAT3* mutation showed similarly low levels of ANC along with low levels of VAF (Fig. 3b), suggesting that even the low mutational burden of *STAT3* could sufficient to inhibit myelopoiesis (Fig. 4). Mariotti et al. suggested that *STAT3* activation interacts with microRNA-146b and then regulates Fas ligand expression to induce neutrophil apoptosis^34^.

Although low levels of ANC have been detected in patients with *STAT3* mutation, the association of *STAT3* mutation with hemoglobin level remains unclear^7,16,33^. Recent study revealed that *STAT3* mutation impaired the increase in erythropoietin-induced phosphorylation of *STAT5* in erythroid precursors. Since the phosphorylation of *STAT5* determines erythropoiesis rate, *STAT3* mutation may induce anemia^35^. In the T-LGL, significantly decreased levels of hemoglobin were detected in the *STAT3* mutant. However, in the T-LGL + PRCA, low levels of hemoglobin were detected regardless of *STAT3* mutation. This finding suggests that combined PRCA by itself can cause a decrease in the level of hemoglobin even in *STAT3* wild type; so that the difference in hemoglobin is obscured by the mutation.

Following *STAT3*, the genes showing frequent mutation in T-LGL included *KMT2D*, *TERT*, *SUZ12*, *BCOR*, *DNMT3A*, and *RUNX1*. Similar to findings reported in previous studies^16,36^, our results showed that the *KMT2D* mutation was the second most common mutation detected in T-LGL. *KMT2D* is regarded as a tumor suppressor, and its mutation leads to induction of lymphomas in mice^37^. A recent study reported on the association of *KMT2D* mutation with risk of lymphoid malignancy in the general population^38^.

Our results showed that four patients had mutations of the *TERT* promoter and showed a good response to treatment. Although the *TERT* gene has been detected in a few cases of aplastic anemia^39^, no study has reported on *TERT* mutation in T-LGL or PRCA even with whole exome sequencing. Application of high depth of sequencing to the proximal promoter region of the *TERT* gene enabled detection of the *TERT* mutation. These hot spot mutations (c.-124C > T and c.-146C > T) have been detected in variable proportions of solid tumors^40^ and in 33% of cases of mantle cell lymphoma^41^. The mutations can lead to increased telomerase activity through transcriptional activation of *TERT*, facilitating telomere maintenance and the subsequent tumorigenesis in human cancers^42^. In addition, the *STAT3* gene can act on the 5’ end of the *TERT* promoter, leading to activation of *TERT*^40,43^. Considering that the VAF levels detected in the *TERT* promoter mutations (patient numbers 4, 9) were respectively similar to those of *STAT3*, the *TERT* mutation clone may be the same as the *STAT3* clone and both mutations may exert a synergistic effect on telomerase activity. Of note, four patients with *TERT* mutations showed good responses to treatments, suggesting that *TERT* mutation-induced florid expansion of T cells may be a sensitive target for immunosuppressants^44^. However, one study reported contrasting results that showed poor prognosis in patients with mantle cell lymphoma, thus conduct of further study will be required^41^.

An association of *TNFAIP3*, another gene showing recurrent mutation in T-LGL, with mutation of *STAT3* has been reported^16,45^. In these papers, the *TNFAIP3* mutations co-occurred with *STAT3* mutations were mostly nonsense or frameshift mutations (8/9, 88.9%). In our results, one nonsense and one frameshift *TNFAIP3* mutations were detected in *STAT3* mutants with VAF levels that were comparable or lower than those of *STAT3*, implying either co-presence as the same clone with *STAT3* or subclone. *TNFAIP3* encodes a regulator of nuclear factor kappa B (NF-kB) signaling; this mutation made truncate A20 protein and induce deregulation of NF-kB activity, with induction of the LGL phenotype^45^. In addition, *SUZ12* regulates chromatin modification and the mutation can contribute to proliferation of cancer cell and T cells^46^. *NF1* is a negative regulator of the Ras signaling pathway and the loss of function mutation results in cell proliferation. Considering that *STAT3* activation in *KRAS* mutant pancreatic cancer was reported to facilitate cell progression^47^, the *KRAS* L19F mutation in our patient (patient number 10) may have interaction potential with *STAT3* mutation. *TP53* mutations could induce T cell proliferation through inhibition and apoptosis. In our result, *DNMT3A* mutations were detected exclusively in the *STAT3* mutant, consistent with the previous report^16^.

Mutations of five genes (*BCOR*, *BCORL1*, *PIGA*, *DNMT3A*, and *ASXL1*) have been detected in 5~10% of cases of aplastic anemia^17^. Because the subclinical T cell clone may be detected in a subset of aplastic anemia, we attempted to examine these mutation frequencies in T-LGL. Contrary to our expectation, no mutation was detected in *BCORL1*, *PIGA*, and *ASXL1*, and only three mutations each were detected in *BCOR* and *DNMT3A*, respectively. The mutation spectrum of T-LGL is considered to differ from that of aplastic anemia.

T-LGL has been detected in 2.5~27% of cases of MDS^12,48^ and MDS has been detected in 5.4% of cases of T-LGL^19^. Our study showed similar results, where MDS was detected in 7.3% (3/41) of cases of T-LGL. There is an expectation that performance of high depth sequencing will lead to discovery of the existence of subclinical MDS. It is unclear whether clonal expansion of T cells occurs as a result of an immune surveillance response to an aberrant MDS clone in already existing MDS or MDS occurs as T-cell mediated DNA damage to myeloid cells and subsequent acquisition of somatic mutations in already existing T-LGL^19,20^. Considering the low levels of VAF detected in many mutations in three patients with T-LGL with concomitant MDS, emergence of a small MDS clone in already existing T-LGL is plausible.

According to our results, among 591 patients who were not diagnosed with T-LGL, *STAT3* mutation was detected in one patient in whom subclinical T-LGL was detected later. Qu et al. reported similar results demonstrating that *STAT3* mutations were predominantly detected in patients with T-LGL, except for six patients with unexplained cytopenia showing low levels of VAF *STAT3* mutations, suggesting the potential for eventual development of T-LGL^30^. Although other study has reported on detection of *STAT3* mutations in a subset of myeloid neoplasms, the authors acknowledged that subclinical T-LGL could not be excluded because further testing for detection of the subclinical T-LGL was not performed^11^.

According to our results, *STAT5B* mutations were detected in one patient with MDS and one patient with hypereosinophilia. Of note, a *STAT5B* N642H mutation was detected in the patient with hypereosinophilia, which was reported to show an association with myeloid neoplasm with eosinophilia^49^. Unlike *STAT3*, *STAT5B* mutations are not specific to T-LGL, and can be detected in other myeloid neoplasms^30^.

*STAT3* mutation was not detected in a substantial fraction of cases of T-LGL. Considering that constitutive activation of *STAT3* was observed even in *STAT3* wild type, clonal expansion of T cells may be induced by other abnormalities such as a mechanism of dysregulation by activated interleukin (IL)-6 and a STAT3 loop in wild type^8^. Interestingly, STAT3 phosphorylation is enhanced by several inflammatory cytokines such as IL-6, IL-15 and MCP-1^50^. Of note, overexpression of IL-15 could contribute to cell growth, chromosomal instability and leukemic transformation of T-LGL^51^.

This study has several limitations. First, evaluation of the characteristics of T-LGL subtypes according to cell phenotype could not be performed due to lack of access to flow cytometry and a relatively low number of patients, thus further study will be required. Second, matched germline samples could not be utilized due to the retrospective design of the study. However, we made a sincere effort to exclude germline variants during data processing. Third, because our data were acquired from one tertiary hospital, there is a possibility of ethnic differences and selection biases for T-LGL and PRCA.

To the best of our knowledge, this is the first study to report on T-LGL + PRCA showing unique features (low level of VAF in the *STAT3* mutation, low level of ALC, and old age), reflecting late emergence of T-LGL in patients with already existing PRCA. In the T-LGL alone subgroup, a low level of ANC was detected in both the *STAT3* mutant and wild type and lower level of hemoglobin was detected in the *STAT3* mutant compared with wild type, while in the T-LGL + PRCA subgroup, low level of hemoglobin was detected regardless of *STAT3* mutation and a low level of ANC was detected in the *STAT3* mutant with a low level of VAF, suggesting that even a low mutational burden of *STAT3* is sufficient to reduce the level of ANC. Beside mutation of *STAT3*, genes showing frequent mutation in T-LGL included *KMT2D* (17.1%), *TERT* (12.2%), *SUZ12* (9.8%), *BCOR* (7.3%), *DNMT3A* (7.3%), and *RUNX1* (7.3%). Concomitant MDS was detected in 7.3% (3/41) of cases of T-LGL through a high depth of NGS. Genes showing recurrent mutation (*BCORL1*, *PIGA*, and *ASXL1*) detected in aplastic anemia were not detected in patients with T-LGL. Of note, the results showed recurrent detection of mutations of the *TERT* promoter with good response to treatment. In addition, *STAT3* mutations were not detected in other myeloid neoplasms, while *STAT5B* mutations were detected in 0.34% of our cohort of patients with hematologic diseases.

In conclusion, the findings of our study suggest that T-LGL+PRCA is a unique disease entity that can be classified as a subtype of T-LGL. Although mutation of *STAT3* leads to a reduction in the levels of ANC and hemoglobin, the underlying severity of T-LGL or PRCA can obscure the reduction effects. Routine utilization of high depth NGS testing can provide sensitive detection of concomitant MDS in patients with T-LGL. *STAT3* mutations suggest the presence of subclinical T-LGL in hematologic diseases. Mutation of the *TERT* promoter may indicate a good response to treatment of T-LGL, thus its addition to the NGS panel may be recommended.

## Methods

### Patient selection

Patients diagnosed with T-LGL (n = 41) or isolated PRCA (n = 3) were enrolled in the study. Patients with T-LGL were divided into two subgroups; T-LGL alone (n = 25) and T-LGL + PRCA (n = 16). T-LGL in our study was diagnosed when the following 3 criteria were met^52^. First, increased number of large granular lymphocytes (> 2 × 10^9^/L) was identified in peripheral blood smear. Second, T cell expansion was confirmed by immunohistochemistry (CD3, CD4, CD8, and granzyme B) of BM biopsy. Third, TCR gene rearrangement was present. If the circulating large granular lymphocyte number was less than 2 × 10^9^/L, both T cell expansion in immunohistochemistry of BM and presence of TCR gene rearrangement along with cytopenia were mandatory. In TCR gene rearrangement, 34 patients (77.3%) were positive for both TCR beta and gamma, 3 patients (6.8%) were positive for TCR beta alone, 4 patients (9.1%) were positive for TCR gamma alone. 3 PRCA patients (6.8%) were negative for both TCR gene rearrangement. Subclinical T-LGL was arbitrarily defined with clinically unsuspected but presence of clonal T cell expansion with *STAT3* mutation^12^. A diagnosis of PRCA was made when anemia (hemoglobin < 10.0 g/dL) with absolute reticulocyte count < 10.0 × 10^9^/L or BM proerythroblasts and/or basophilic erythroblasts < 5% of total nucleated cells^4^.

In addition, a review of 591 patients (173 with acute myeloid leukemia, 96 patients with MDS, 85 patients with myeloproliferative neoplasm (MPN), 77 patients with acute lymphoblastic leukemia, 35 patients with plasma cell myeloma, 28 patients with chronic lymphocytic leukemia, 20 patients with MDS/MPN, and 77 patients with cytopenia caused by other factors) who were requested for an NGS panel of hematologic malignancies at Seoul National University Hospital was conducted for identification of *STAT3* and *STAT5B* mutation frequencies in hematologic diseases without T-LGL. These studies were approved by the institutional review board of Seoul National University Hospital (H-2106–128-1228) and were conducted in accordance with the Declaration of Helsinki. Informed consent was obtained from all patients.

### Evaluation of bone marrow dysplasia and T cell clonality

An independent review of BM dysplasia was performed by two hematopathologists for detection of MDS. MDS based on criteria established by the WHO^52^ (≥ 10% dysplasia of ≥ 1 lineage). MDS was confirmed when both hematopathologists reached agreement. Anti-CD3 (DAKO, USA), CD20 (DAKO), CD4 (Leica biosystem, USA), CD8 (Roche, Switzerland), granzyme B (Roche), and CD56 antibodies (Roche), EBER in-situ hybridization (Ventana ISH iView Blue Detection Kit, Roche) were used with automatic staining (Ventana Benchmark Ultra, Roche) for exclusion of other mature T cell neoplasm and assessment of T cell expansion in BM. Most of T-LGL patients (36/41, 87.8%) expressed CD8, 2 patients (4.9%) expressed CD4, 1 patient (2.4%) were CD4/8 coexpressed and the remaining 1 patient (2.4%) showed undetermined phenotype due to poor stainability. Analysis of rearrangements of TCR gene beta and gamma chain was performed using the BIOMED-2 assay (Invivoscribe, USA) according to the manufacturer’s instructions.

### Custom targeted NGS panels and data processing

Two custom targeted NGS panels were used. First, 84 genes including genes reported to show mutation in PRCA or aplastic anemia^7,17,18,23–25^, genes showing recurrent mutation in myeloid hematologic malignancy and *STAT3* and *STAT5B* were used for T-LGL and/or PRCA patients (Supplementary Table 1). Sequencing was performed using NovaSeq6000 (Illumina, USA) by whole blood of BM (n = 22) or peripheral blood (n = 22) according to the manufacturer’s instructions. Ten unrelated healthy blood specimens without evidence of hematologic malignancy were used as controls. Analysis of data was performed using an in-house bioinformatics pipeline (Supplementary Method). The median depth of coverage was 1502 x (interquartile range, IQR; 1170–2204) and the lowest depth of coverage was 811 × for all samples. The cut-off for reporting variants was set as ≥ 2% VAF.

Second, a panel of 103 hematologic malignancy-related genes including *STAT3* and *STAT5B* was used for hematologic diseases without T-LGL (Supplementary Table 1). Sequencing was performed using NextSeq550Dx (Illumina, USA) along with preparation of the DNA and RNA library using Archer VariantPlex Somatic Reagents and Archer FusionPlex Kits (ArcherDx Inc., USA). Analysis of sequence data was performed using Clinical Genomics Workspace (PierianDX Inc., USA). The average depth of coverage was > 200 × and the cut-off value was set as ≥ 2% VAF for all samples.

### Treatment response

In our retrospective analysis for 44 patients, 31 patients who were symptomatic, transfusion-dependent, or had worsening of hematological parameters were treated with a various regimens including corticosteroid, methotrexate, cyclophosphamide, and/or cyclosporine (Supplementary Table 6). 13 patients were not treated based on asymptomatic, transfusion-independent, or clinical decision. Evaluation of treatment responses was performed according to the previous report with some modification^53^. The RBC transfusion dependency was defined as receiving more than three RBC transfusions within 16 weeks with at least two transfusion episodes. Complete response (CR) was defined as no need for RBC transfusion, ANC > 1.8 × 10^9^/L, platelet > 100 × 10^9^/L and lymphocytosis < 4.0 × 10^9^/L during the treatment period of 16 to 24 weeks. Partial response (PR) was defined as a reduction of RBC transfusion by > 50%, ANC > 1.0 × 10^9^/L, but < 1.8 × 10^9^/L, platelet > 50 × 10^9^/L, but < 100 × 10^9^/L. Patients who did not meet these criteria were defined as no response (NR).

### Statistical analysis

Chi-square test or Fisher's exact test and Wilcoxon rank sum test were performed for comparison of subgroups. Spearman correlation was performed to determine the association of the extent of *STAT3* mutation with CBC parameters. *P* < 0.05 was considered statistically significant. All statistical analyses were performed using R version 4.2.1.

## Supplementary Information

Supplementary Information 1.

Supplementary Information 2.

Supplementary Information 3.

## Data Availability

The raw fastq files of this study have been submitted for the Sequence Read Archive (SRA) repository with the bioProject PRJNA919192.
