# Supplementary material for: Distinct mutational pattern of T-cell large granular lymphocyte leukemia combined with pure red cell aplasia: low mutational burden of STAT3
Source: Sci Rep. 2023 May 4;13:7280. doi: 10.1038/s41598-023-33928-z (PMC10160083; doi:10.1038/s41598-023-33928-z)
Supplement: Supplementary file 1 — Supplementary Information 1. [file 41598_2023_33928_MOESM1_ESM.docx]

**Title: Distinct mutational pattern of T-cell large granular lymphocyte leukemia combined with pure red cell aplasia: Low mutational burden of *STAT3***

Sooyong Park^1^, Jiwon Yun^2^, Sung Yoon Choi^1^, Dajeong Jeong^1^, Ja-Yoon Gu^1,3^, Jee-Soo Lee^1^, Moon-Woo Seong ^1^, Yoon Hwan Chang^1^, Hongseok Yun^4*^, Hyun Kyung Kim^1,3*^

^1^Department of Laboratory Medicine, Seoul National University College of Medicine, Seoul, Republic of Korea. ^2^Department of Laboratory Medicine, Chung-Ang University College of Medicine, Seoul, Republic of Korea. ^3^Cancer Research Institute, Seoul National University College of Medicine, Seoul, Republic of Korea. ^4^Department of Genomic Medicine, Seoul National University Hospital, Seoul, Korea. *E-mail: hs.yun@sun.ac.kr; lukekhk@snu.ac.kr

**Supplementary Information**

**Supplementary Method.** In-house bioinformatics analysis of targeted NGS data for T-LGL and/or PRCA cohort (page 3)

**Supplementary Figure 1.** Lollipop plot of signal transducer and activator of transcription 3 (*STAT3*) gene (page 4)

**Supplementary Figure 2.** Proportions of signal transducer and activator of transcription 3 (*STAT3*) mutation type in terms of variant allele frequency (VAF) of *STAT3* (page 5)

**Supplementary Table 1.** Two custom targeted sequencing panels (page 6-7)

**Supplementary Table 2.** Mutations identified in three T-LGL patients concomitant MDS (page 8)

**Supplementary Table 3.** Clinical characteristics of three T-LGL patients concomitant MDS (page 8)

**Supplementary Table 4.** 65 mutations of 28 genes identified in 32 patients with T-LGL or T-LGL+PRCA (page 8)

**Supplementary Table 5.** List of genes (n=56) in which no mutation was detected in patients with T-LGL or T-LGL+PRCA (page 9)

**Supplementary Table 6.** Clinical characteristics of 31 treated patients (page 10)

**Supplementary Method.** In-house bioinformatics analysis of targeted NGS data for T-LGL and/or PRCA cohort

Analysis of the FASTQ files generated from the customized panel sequencing was performed using the SNUH FiRST Panel Analysis Pipeline. The alignment to the hg19 reference genome was performed using BWA-mem (v0.7.17) and GATK Best Practice. Variants including single-nucleotide variants (SNVs) and small insertions-deletions (InDels) were subsequently identified using HaplotypeCaller, SNVer, and LoFreq. Annotation of the detected variants was then performed using SnpEff (v5.0) and various databases including RefSeq, COSMIC (v96), dbSNP (build 155), ClinVar (last accessed Nov 13, 2022), and gnomAD (v2.1.1). To identify and remove possible germline variants, SNVs and InDels commonly detected (≥0.1%) in population databases such as the 1000 genomes project version and gnomAD were filtered for detection of possible germline variants. In addition, mutations detected in healthy controls and most mutations that showed 40~60 % VAF were excluded as potential germline mutations.


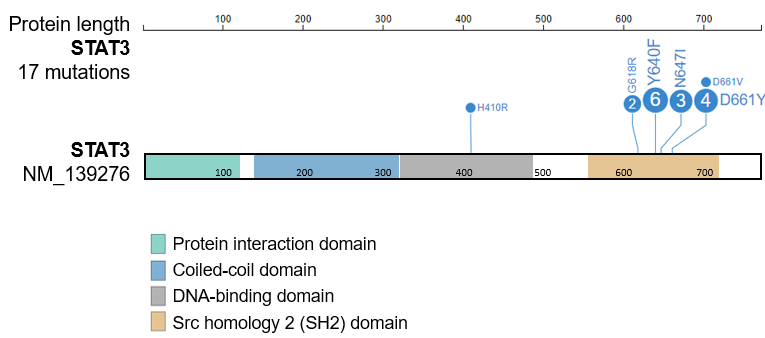


**Supplementary Figure 1.** Lollipop plot of signal transducer and activator of transcription 3 (*STAT3*) gene. Of total 17 *STAT3* mutations detected, 94.1% (16/17) were located in the src homology 2 domain. Annotations for each domain were described at the bottom.

**
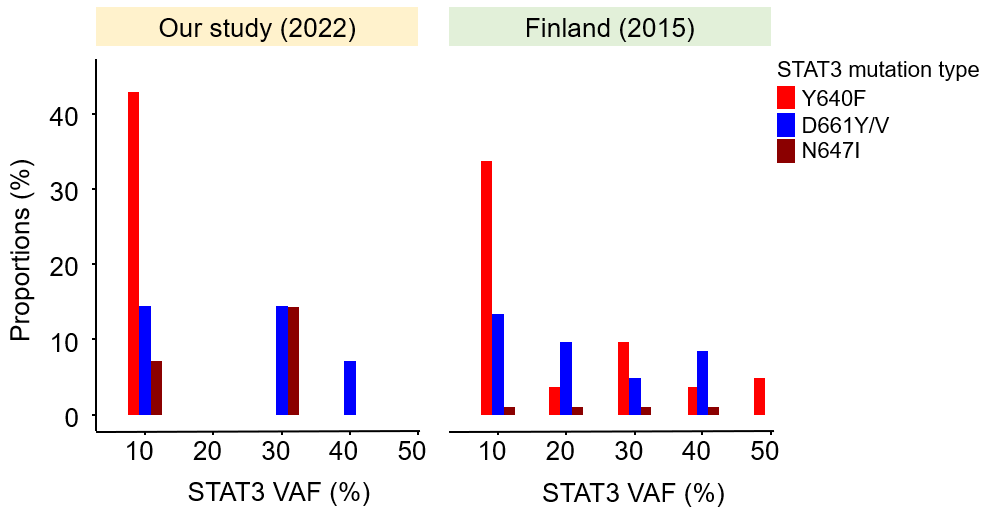
**

**Supplementary Figure 2.** Proportions of signal transducer and activator of transcription 3 (*STAT3*) mutation type in terms of variant allele frequency (VAF) of *STAT3*. The levels of VAF were stratified according to each 10% increment. Results of our study and those of the Finland study (Rajala *et al*, Haematologica, 2015;100:91-9) are shown together. The most common mutation was Y640F marked in the red bar in both studies. The Y640F showed highest proportions in <10% VAF of *STAT3* mutation both in our study (66.7%, 6/9) and in the Finland study (59.6%, 28/47).

**Supplementary Table 1.** Two custom targeted sequencing panels

| Panel including mutated genes  in PRCA or aplastic anemia or myeloid neoplasm (n=84)^a^ | | Hematologic malignancies-related  panel (n=103)^b^ | |
| --- | --- | --- | --- |
| ABL1 | MPL | APC | ABL1 (hot spot) |
| ASXL1 | MYD88 | ARID1B | ANKRD26 (hot spot) |
| ATM | NOTCH1 | ARID2 | ATRX (hot spot) |
| ATRX | NF1 | ASXL1 | BIRC3 (hot spot) |
| BCOR | NPM1 | ATM | BRAF (hot spot) |
| BCORL1 | NRAS | B2M | BTK (hot spot) |
| BRAF | PDGFRA | BCOR | CALR (hot spot) |
| BRCC3 | PEG3 | BCORL1 | CBL (hot spot) |
| CALR | PHIP | CARD11 | CBLB (hot spot) |
| CBL | PHF6 | CCND1 | CBLC (hot spot) |
| CBLB | PIGA | CD28 | CCND2 (hot spot) |
| CBLC | POT1 | CD58 | CDC25C (hot spot) |
| CDKN2A | PPM1D | CD79A | CDKN2A (hot spot) |
| CEBPA | PRPF8 | CHD8 | CEBPA (hot spot) |
| CSF3R | PTEN | CREBBP | CSF3R (hot spot) |
| CSMD1 | PTPN11 | CUX1 | CXCR4 (hot spot) |
| CUX1 | RAD21 | DDX41 | DCK (hot spot) |
| DIS3 | RAP1A | DIS3 | DHX15 (hot spot) |
| DNMT3A | RBBP4 | DNMT3A | ETNK1 (hot spot) |
| EED | RIT1 | EP300 | FLT3 (hot spot) |
| ETV6 | RUNX1 | ETV6 | FOXO1 (hot spot) |
| EZH2 | SETBP1 | EZH2 | GATA1 (hot spot) |
| FBXW7 | SF3B1 | FBXW7 | GNAS (hot spot) |
| FLT3 | SH2B3 | GATA2 | HRAS (hot spot) |
| GATA1 | SLC20A1 | GNA13 | IDH1 (hot spot) |
| GATA2 | SMC1A | IKZF1 | IDH2 (hot spot) |
| GNAS | SMC3 | KDM6A | JAK2 (hot spot) |
| GNB1 | SRCAP | KIT | JAK3 (hot spot) |
| HRAS | SRSF2 | KMT2A | KMT2C (hot spot) |
| IDH1 | STAG2 | KMT2D | KRAS (hot spot) |
| IDH2 | STAT3 | LUC7L2 | MAP2K1 (hot spot) |
| IKZF1 | STAT5B | MYC | MEF2B (hot spot) |
| JAK1 | SUZ12 | NF1 | MPL (hot spot) |
| JAK2 | TERT | NRAS | MYD88 (hot spot) |
| JAK3 | TET2 | PHF6 | NOTCH1 (hot spot) |
| KDM3B | TNFAIP3 | PTEN | NOTCH2 (hot spot) |
| KDM6A | TP53 | RAD21 | NPM1 (hot spot) |
| KIT | U2AF1 | RB1 | PDGFRA (hot spot) |
| KMT2A | WT1 | RUNX1 | PPM1D (hot spot) |
| KMT2D | ZRSR2 | SH2B3 | PTPN11 (hot spot) |
| KRAS | MSR1 | SMC1A | RBBP6 (hot spot) |
| LAMB4 | HOXC9 | SMC3 | RHOA (hot spot) |
|  |  | SRSF2 | RPS14 (hot spot) |
|  |  | STAG2 | SETBP1 (hot spot) |
|  |  | TET2 | SF3B1 (hot spot) |
|  |  | TNFAIP3 | SLC29A1 (hot spot) |
|  |  | TNFRSF14 | STAT3 (hot spot) |
|  |  | TP53 | STAT5B (hot spot) |
|  |  | TRAF3 | U2AF1 (hot spot) |
|  |  | U2AF2 | XPO1 (hot spot) |
|  |  | WT1 |  |
|  |  | ZAP70 |  |
|  |  | ZRSR2 |  |

^a^ 84 genes included genes reported to be once mutated in PRCA or aplastic anemia or recurrently mutated genes in myeloid neoplasm in terms of the following six studies reported in the literatures (Sci Rep 2021;11:1-7, Ann Hematol 2020;99:1749-1754, N Engl J Med 2012;366:1905-13, N Engl J Med 2015;373:35-47, Lancet Oncol 2017;18:100-111, Biol Blood Marrow Transplant 2019;25:2517-2521).

^b^ 103 genes known as recurrent mutated genes in hematologic malignancies were designed in the clinical laboratory at Seoul National University Hospital.

**Supplementary Table 2.** Mutations identified in three patients with T-LGL concomitant MDS

All mutations are listed in separated files

“Supplementary_table_mutation_lists.xlsx”

**Supplementary Table 3.** Clinical characteristics of three T-LGL patients concomitant MDS

| Patient number | 15 | 36 | 44 |
| --- | --- | --- | --- |
| Age (years) | 75 | 74 | 57 |
| Gender | Female | Female | Female |
| Hemoglobin (g/dL) | 3.8 | 8.2 | 6.1 |
| WBC (x 10^9^/L) | 2.44 | 5.89 | 3.18 |
| ANC (x 10^9^/L) | 1.14 | 4.24 | 1.53 |
| Platelet (x 10^9^/L) | 184 | 117 | 231 |
| Chromosome | 46,XX | 46,XX | 46,XX |
| Bone marrow dysplasia | Dysgranulopoiesis | Dyserythropoiesis | Dysgranulopoiesis |

**Supplementary Table 4.** 65 mutations of 28 genes identified in 32 patients with T-LGL or T-LGL+PRCA

All mutations are listed in separated files

“Supplementary_table_mutation_lists.xlsx”

**Supplementary Table 5.** List of genes (n=56) in which no mutation was detected in patients with T-LGL or T-LGL+PRCA

| ABL1 | KIT |
| --- | --- |
| ASXL1 | MPL |
| ATRX | MYD88 |
| BCORL1 | NOTCH1 |
| BRAF | NRAS |
| BRCC3 | PDGFRA |
| CALR | PEG3 |
| CBL | PHF6 |
| CBLB | PHIP |
| CDKN2A | PIGA |
| CSF3R | POT1 |
| DIS3 | PPM1D |
| ETV6 | PRPF8 |
| EZH2 | PTPN11 |
| FBXW7 | RAD21 |
| FLT3 | RAP1A |
| GATA1 | RBBP4 |
| GATA2 | RIT1 |
| GNAS | SETBP1 |
| GNB1 | SF3B1 |
| HOXC9 | SLC20A1 |
| HRAS | SMC1A |
| IDH2 | SMC3 |
| IKZF1 | SRCAP |
| JAK1 | SRSF2 |
| JAK2 | TET2 |
| JAK3 | WT1 |
| KDM3B | ZRSR2 |

**Supplementary Table 6.** Clinical characteristics of 31 treated patients

Clinical characteristics are listed in separated files

“Supplementary_table_6.xlsx”
